# Anthracycline-induced hypertension in pediatric cancer survivors: unveiling the long-term cardiovascular risks

**DOI:** 10.1186/s43044-024-00506-1

**Published:** 2024-06-07

**Authors:** Andia Taghdiri

**Affiliations:** Faculty of Medicine, Ivane Javakhishvili Tbilisi State University, Tbilisi, Georgia

**Keywords:** Anthracycline, Hypertension, Anthracycline-induced hypertension, Anthracycline-induced cardiotoxicity, AIC, Cardiotoxicity, Cancer survivor-ship, Long-term cardiovascular risk, Survivor care strategies, Pediatric oncology

## Abstract

**Background:**

Long-term cardiovascular complications are common among pediatric cancer survivors, and anthracycline-induced hypertension has become an essential reason for concern. Compared to non-cancer controls, survivors have a higher prevalence of hypertension, and as they age, their incidence rises, offering significant dangers to cardiovascular health.

**Main body:**

Research demonstrates that exposure to anthracyclines is a major factor in the development of hypertension in children who have survived cancer. Research emphasizes the frequency and risk factors of anthracycline-induced hypertension, highlighting the significance of routine measurement and management of blood pressure. Furthermore, cardiovascular toxicities, such as hypertension, after anthracycline-based therapy are a crucial be concerned, especially for young adults and adolescents. Childhood cancer survivors deal with a variety of cardiovascular diseases, such as coronary artery disease and cardiomyopathy, which are made worse by high blood pressure. In order to prevent long-term complications, it is essential to screen for and monitor for anthracycline-induced hypertension. Echocardiography and cardiac biomarkers serve as essential tools for early detection and treatment. In order to lower cardiovascular risks in pediatric cancer survivors, comprehensive management strategies must include lifestyle and medication interventions in addition to survivor-centered care programs.

**Short conclusion:**

Proactive screening, monitoring, and management measures are necessary for juvenile cancer survivors due to the substantial issue of anthracycline-induced hypertension in their long-term care. To properly include these strategies into survivor-ship programs, oncologists, cardiologists, and primary care physicians need to collaborate together. The quality of life for pediatric cancer survivors can be enhanced by reducing the cardiovascular risks linked to anthracycline therapy and promoting survivor-centered care and research.

## Background

Over the past several decades, childhood cancer survival rates have considerably increased, particularly in high-income nations where more than 80% of cancer patients live for 5 years or more following diagnosis [1, 2]. The World Health Organization estimates that 400,000 children and adolescents between the ages of 0 and 19 years will get cancer each year [3, 4].

Survivors of childhood cancer may confront distinct challenges after receiving their first diagnosis and treatment, as cancer therapy frequently leads to long-term health complications [5]. Compared to matched controls, long-term survivors had three times the prevalence of severe chronic health conditions [6]. An important issue among these side effects is anthracycline-induced hypertension (AIH), which calls for aggressive screening, monitoring, and management techniques to lessen its negative effects on the cardiovascular health of survivors [7]. In a study by Pearlstein et al. [8], 13,266 cancer survivors with hypertension had a prevalence of nearly 53.2%.

Doxorubicin and daunorubicin (DNR), two anthracyclines with strong anti-tumor properties, are essential in pediatric oncology [9, 10]. But the administration of these drugs entails a high risk of cardiotoxicity, which can show up as a variety of cardiovascular problems, from acute arrhythmias to chronic heart failure, that may appear months or even years after therapy ends [11–13]. The most pernicious of these side effects is hypertension, which exacerbates the cardiomyopathy brought on by anthracycline and increases cardiovascular morbidity, ultimately decreasing the quality of life for survivors [7, 11–13].

Chronic hypertension exerts stress on the heart, which can result in cardiac weakness and thickening and eventually heart failure [14]. Simultaneously, it promotes arterial constriction via cholesterol accumulation, increasing the effort and decreasing the effectiveness of the circulatory system [14, 15]. This narrowing of the arteries, known as atherosclerosis, decreases coronary blood flow and increases the risk of atherosclerotic cardiovascular disease (ASCVD) in both the general population and cancer patients [16, 17].

Effective screening and therapy of AIH require an understanding of its pathogenesis. The formation of reactive oxygen species, mitochondrial dysfunction, and disturbance of iron homeostasis are only a few of the processes by which anthracyclines cause cardiotoxicity, which in turn causes myocardial damage and dysfunction [18]. The risk of AIH is further increased by variables including cumulative dose, gender, genetic susceptibility, and concomitant exposure to other cardiotoxic drugs such radiation or trastuzumab [18].

It is difficult to identify hypertension as a crucial consequence for survivors of anthracycline-induced cardiotoxicity (AIC). The medical profession is still split on the best method for screening and monitoring despite the evident link between anthracycline treatment and hypertension [19]. Incorporating hypertension control into long-term health initiatives necessitates the use of survivor-ship care plans (SCPs) [20, 21]. Despite this significance, there are still a number of important information gaps, such as those pertaining to screening techniques, the efficacy of pharmacological therapies, and the significance of lifestyle modifications [11, 22]. Comparative study is further hampered by inconsistent definitions and reporting of AIH [11, 22].

The aim of this narrative review is to describe the particular cardiovascular risks that children who have survived cancer face, especially the risk of AIH. It draws attention to the increasing demand for early detection, evidence-based intervention strategies, and proactive cardiovascular screening that are customized to meet the specific requirements of this population. The study also intends to encourage more research on the cardiotoxic effects of anthracycline therapy, therefore advancing improved clinical practices that enhance long-term survivor-ship care.

## Main body

### Anthracycline-induced cardiotoxicity

Acute, subacute, and chronic forms of AIC are distinguished by their unique features and symptoms [17].

Acute cardiotoxicity, typically observed immediately after injection, often presents as myocarditis, arrhythmias, or abnormalities on electrocardiograms [10, 23]. This variety of irregular heartbeats and abnormalities on the electrocardiogram (ECG) can be mild to potentially fatal [10, 23]. A frequent arrhythmia called sinus tachycardia, which is characterized by a heart rate that is higher than usual at rest, might be a result of myocardial damage-related stress response or compensatory mechanism [22]. Although less prevalent in children, atrial fibrillation can happen after anthracycline medication and is characterized by fast and irregular heartbeats [22]. Serious side effects from the ventricles, such as ventricular fibrillation and rapid cardiac death, can result from ventricular tachycardia [17].

Changes in the ST-T waves in relation to ECG anomalies may suggest myocardial ischemia or injury [22, 24]. Changes during the repolarization phase of the cardiac cycle, seen as ST depression or T wave inversion on an ECG, might be the consequence of anthracycline-induced cardiotoxicity [22, 24]. Torsades de pointes, a potentially fatal kind of ventricular tachycardia, can occur more frequently in patients with prolonged QT intervals, which lengthen the delay between the beginning of the Q wave and the end of the T wave in the heart's electrical cycle [22, 24]. Furthermore, myocardial injury or situations where there is an enlargement of the space between the heart and the chest wall—such as in pericardial effusion, a consequence of myocarditis—may also be associated with low QRS voltage [22, 25].

On the other hand, subacute cardiotoxicity usually appears within a year of starting therapy. It can appear as pericarditis–myocarditis syndrome, which is characterized by inflammation of the heart or its surrounding sac and results in symptoms like chest pain, shortness of breath, and fluid accumulation around the heart [17, 23].

Chronic cardiotoxicity may end up in left ventricular (LV) dysfunction, congestive heart failure, and dilated cardiomyopathy (DCM). It can happen months or even years after medication and is frequently irreversible [17, 23]. Palpitations, fatigue, peripheral edema, and dyspnea are possible signs of persistent cardiotoxicity [18]. According to a study by Alkofide et al. [19], out of 235 cancer patients who received anthracycline treatment, 8.9% showed signs of chronic cardiotoxicity and 28.9% had acute cardiotoxicity.

The severity and likelihood of AIC are influenced by a number of factors.

First of all, the risk of cardiotoxicity is correlated with the total cumulative dose of anthracyclines delivered; greater cumulative doses are linked to higher rates of cardiac events [18]. For instance, the rates of cardiac events associated with anthracycline therapy are 7%, 18%, and 65%, respectively, at cumulative doses of 150, 350, and 550 mg/m^2^ [18].

Additionally, a major factor influencing the risk of cardiotoxicity is the time and frequency of anthracycline treatment. When compared to larger, less frequent doses, administering anthracyclines in smaller, more frequent doses may reduce the risk of cardiotoxicity and help the heart recover from the inflammation and oxidative stress that these drugs cause [26].

Furthermore, patient-specific variables that affect sensitivity to AIC include age and comorbidities [18, 26]. Compared to middle-aged people, younger patients (< 18 years) and older patients (> 65 years) are more susceptible to cardiotoxicity, most likely because of differences in heart development, metabolism, and repair mechanisms [18, 26].

Also, genetic factors are important in determining how susceptible and responsive an individual is to AIC. Certain genetic variants affect the anthracycline metabolism, transport, and detoxification, and they also affect the creation and function of cardiac proteins, which might affect an individual's vulnerability to cardiotoxicity [26, 27].

### Pathophysiology of AIC and hypertension

Effective management and mitigation of these deleterious consequences, with hypertension standing out as a critical result of AIC, need an understanding of the complex biology underlying AIC and related hypertension. The mechanisms involved in these processes are complex and encompass several facets of the anatomy and function of the heart.

#### Apoptosis and inhibition of topoisomerase 2β

Anthracyclines are the main reason for cardiotoxicity because they coordinate cellular mechanisms that are mainly concerned with apoptosis and topoisomerase 2β inhibition [18]. By binding to and blocking topoisomerase 2β, an essential enzyme that controls transcription and DNA structure, anthracyclines induce DNA damage, activate cell death pathways, and reduce mitochondrial biogenesis [18]. The clinical risk of heart failure linked to anthracycline exposure is increased by this specific inhibition of cardiomyocytes [18].

Concurrently, anthracyclines cause DNA breakage and caspase activation in cardiomyocytes and endothelial cells, which results in programmed cell death, or apoptosis [28, 29]. Since anthracyclines suppress topoisomerase 2β, pro-apoptotic proteins like p53 are further activated, which results in endothelial dysfunction, vascular integrity problems, and decreased cardiac contractility [28, 29]. Moreover, nitric oxide (NO) synthesis decreases and arterial integrity deteriorates, leading to stiffened and constricted blood arteries, which raise blood pressure [29].

#### Oxidative stress

A number of cardiovascular health-harming processes, such as iron chelation, redox cycling, and mitochondrial dysfunction, are involved in anthracycline-induced oxidative stress [28, 30]. Inflammation, apoptosis, necrosis, and autophagy in cardiomyocytes are brought on by reactive oxygen species (ROS) produced by these processes, which harm cellular constituents and impair antioxidant defense mechanisms [28, 30]. The resultant endothelial dysfunction reduces the generation and bioavailability of NO, which in turn causes vascular constriction and hypertension [28, 31].

#### Endocrine dysfunction

Anthracyclines interfere with the function of endocrine organs, including the thyroid, pituitary, and adrenal glands, that regulate blood pressure [18, 28].

Hypertension and anthracycline-induced hypothyroidism have a complicated interaction. Conversely, there are a number of ways in which hypothyroidism can exacerbate hypertension [18, 28]. One of the main causes of the observed cardiovascular changes is the increase in peripheral vascular resistance caused by vascular systemic alterations associated with hypothyroidism, such as reduced compliance and increased artery wall stiffness [18, 28, 32]. Similar to this, anthracycline exposure results in hypercortisolism, which raises renin–angiotensin–aldosterone system (RAAS) activity and causes RAAS-mediated hypertension as well as increased reabsorption of salt and water [18, 28].

Reduced oxygen and substrate consumption by the body's primary organ systems is another effect of hypothyroidism. As a result, less cardiac output is required. Furthermore, by changing the expression of genes unique to myocytes, hypothyroidism directly affects cardiac function by affecting heart muscle cells [33]. Increased peripheral vascular resistance, lowered heart rate, decreased cardiac output, and impaired contractility are the main circulatory abnormalities linked to hypothyroidism [33]. Tissue oxygen consumption decreases in tandem with this [33]. On the other hand, hypertension may occur as a result of the hypothyroidism-induced rise in peripheral vascular resistance, which can offset the decreased cardiac output [32]. This is especially important for young cancer survivors since their cardiovascular systems could be compromised as a result of their cancer therapy.

#### Nephrotoxicity

Anthracyclines induce damage to the kidneys, including tubular atrophy and proteinuria, which throws off the body's fluid and electrolyte balance as well as the RAAS [18, 28]. Hypertension develops as a result of dysregulated RAAS, reduced NO generation, and salt and water retention [18, 28].

#### Fibrosis

Treatment with anthracyclines activates fibroblasts and myofibroblasts, which causes the heart and artery walls to deposit excessive amounts of collagen [28, 31]. This mechanism ultimately leads to hypertension by increasing vascular and myocardial stiffness [28, 31].

Anthracyclines induce inflammation and immune response activation, which in turn causes the release of cytokines and chemokines, drawing immune cells to the heart and promoting cardiac fibrosis, with additional cardiotoxic consequences [18]. Furthermore, anthracycline-induced mitochondrial dysfunction worsens oxidative stress and hypertension by interfering with the synthesis of adenosine triphosphate, cardiac contractility, and NO availability [34–36]. In addition, by producing arterial damage, inflammation, and platelet activation, anthracyclines increase the risk of thrombosis and exacerbate cardiovascular health issues [35]. Also, anthracyclines cause cardiac atrophy, which impairs cardiac function and increases the risk of arrhythmias and ischemia [37]. Sarcoplasmic reticulum dysfunction, another term for disturbance of the calcium handling mechanism in cardiomyocytes, can potentially cause arrhythmias and cardiac dysfunction [38–40]. Ultimately, iron–anthracycline complexes are created when anthracyclines chelate iron, which exacerbates cardiovascular damage by increasing oxidative stress [18].

Early aging, lifestyle variables, and psychological stresses are among the extra hypertension risk pathways that survivors of childhood cancer must contend with [7, 41, 42]. The unique features of AIH, including its early onset and probable reversibility, emphasize how critical early identification and management are in reducing the risk of cardiotoxic consequences [17, 28, 43].

### Prevalence of AIH in pediatric cancer survivors

In a population-based Canadian cohort research involving over 6,000 cancer survivors [44], 43% of cancer survivors and only 31% of the non-cancer controls reported having hypertension. In addition, the prevalence of hypertension increased with age, with almost 70% of survivors experiencing hypertension by the time they were 50 years old, compared to 13% at 30 years old and 37% at 40 years old [44].

#### Prevalence of hypertension in survivors

In a prospective study, Chow et al. [45] investigated the prevalence of hypertension and its associated risk factors in adult cancer survivors who received their diagnosis as children. The prevalence of hypertension was determined to be 2.8%, which was higher than expected rates based on age and sex standards. The history of chronic illness, advanced age, elevated body mass index (BMI), and abdominal radiation were risk factors. In a research by Wong-Siegel et al. [46], the cardiovascular toxicities following vascular endothelial growth factor (VEGF)–targeted and anthracycline-based therapy were examined in cancer survivors who were adolescents and young adults. Of those receiving anthracycline, VEGF inhibitor, or both, they found cardiovascular toxicities in 32%, 22%, and 34% of cases; the most commonly reported side effect was hypertension. Anthracycline therapy-associated cardiovascular toxicities have been linked to male sex as an independent risk factor. In another study by Mulrooney et al. [47], 1,853 patients who had received cardiotoxic therapy for pediatric cancer at least 10 years prior had their heart health evaluated. Among adult survivors, they discovered a range of cardiac abnormalities, including conduction/rhythm issues (4.6%), cardiomyopathy (7.4%), valve disease (28%), and coronary artery disease (3.8%). The prevalence of coronary artery disease and cardiomyopathy was significantly correlated. When the St. Jude Lifetime Cohort evaluation [44] was conducted, 23.3% of the survivors had hypertension. Cardiomyopathy and coronary artery disease were linked to higher probabilities of hypertension. A summary of the studies is presented in Table 1 [45–47].

**Table 1:** Childhood cancer survivors and cardiovascular complications of their cancer therapy: summary of studies

Author	Chow et al. [45]	Wong-Siegel et al. [46]	Mulrooney et al. [47]
No. of population	13,060 Childhood cancer survivors, 4023 CCSS siblings, and 11,817 from external validation cohorts	1165	3054
Participant	Children diagnosed with cancer before 21 year between 1970 and 2001, surviving at least 5 year post-diagnosis	AYA cancer survivors who received anthracycline and/or VEGF inhibitor	1853 Adult survivors of childhood cancer exposed to cardiotoxic therapy
Methodology	Used Poisson and Cox regression to link demographic and treatment factors to heart failure risk by age 40 year	Retrospective study using electronic medical records and cancer registry data	Cross-sectional study assessing health with history, physicals, metabolic panels, echocardiograms, electrocardiograms, and 6-min walk tests
Implications	The models offer personalized risk assessment and screening strategies for childhood cancer survivors	To assess the burden of CT after anthracycline and/or VEGF inhibitor therapy	Systematically evaluate cardiac outcomes in childhood cancer survivors and identify subclinical disease
Sample size	285 CCSS participants and 12 siblings experienced heart failure by age 40 yr	374 Received anthracycline only, 255 received VEGF inhibitor only, and 536 received both	1853 Adults, childhood cancer survivors, completed baseline evaluation in St. Jude Lifetime Cohort
Anthracycline association	Higher anthracycline doses predict increased heart failure risk	Anthracycline increased CT risk, especially in men	Anthracycline exposure ≥ 250 mg/m^2^ raises odds for cardiomyopathy (OR, 2.7; 95% CI, 1.1–6.9) and associates with valvular disease (interaction P < 0.001)
Prevalence of hypertension in survivors	2.8% CCSS survivors had hypertension at baseline (average 15 yr post-diagnosis)	Hypertension was the most common CT (19%–34%)	23.3% Participants had hypertension
Cardiotoxicity	Heart failure, defined as CTCAE grade 3 or higher, necessitating medications, transplant, or causing death	CT was defined as incident hypertension, coronary artery disease, myocardial infarction, cardiomegaly, cardiomyopathy/heart failure, conduction abnormalities, and cerebrovascular events	Exposures: anthracycline and radiation therapy Outcomes: cardiomyopathy, coronary disease, valve function, conduction/rhythm issues
Demographic	47% CCSS survivors were female; median age at last follow-up, 32 yr	Sex, race, age, and baseline comorbidities	Median age at diagnosis: 8.0 yr (range: 0–24 yr), evaluated at 31.0 yr (18–60 yr)
Follow-up duration	CCSS survivors had a median 19-yr follow-up (range: 0–34 yr)	Median follow-up was 1.5, 0.6, and 1.1 yr for anthracycline only, VEGF inhibitor only, and both groups, respectively	Cross-sectional study with data collected during the baseline evaluation
Key finding	Treatment dosages, sex, and diagnostic age are used in predictive models to accurately identify and forecast the risk of heart failure in pediatric cancer survivors. These models categorize survivors into low-, moderate-, and high-risk categories, each of which is linked to a different incidence of heart failure, and have been validated in external cohorts. This helps medical professionals improve at-risk survivor surveillance and intervention techniques	Among AYA cancer survivors treated with anthracycline and/or VEGF inhibitors, CT was often seen. A risk factor for CT following anthracycline was male sex. The individuals who were treated with both VEGF inhibitor and anthracycline had the greatest cumulative incidence of CT. Additional monitoring and screening are required	Adult survivors of childhood cancer may have latent illness discovered by cardiovascular screening. As people age, the prevalence of cardiac problems increases. Cardiomyopathy and coronary artery disease have been related to decreased physical performance

CCSS: Childhood Cancer Survivor Study, AYA: adolescent and young adult, VEGF: vascular endothelial growth factor, CT: cardiovascular toxicity, OR: odds ratio, CI: confidence interval, CTCAE: Common Terminology Criteria for Adverse Events

### Association with anthracycline exposure compared to other cancer treatments

An investigation at the Union Hospital, Tongji Medical College, Huazhong University of Science and Technology (Wuhan, China) [48], discovered that the cumulative dosage of DNR affected the incidence of cardiac disorders (P = 0.030; odds ratio, 1.553; 95% confidence interval, 1.005–3.108). According to another research [49], AIC affects one in 10 children who had cumulative anthracycline doses more than 300 mg/m^2^.

#### Temporal patterns post-treatment

A University Hospitals Seidman Cancer Center study [50] revealed that for patients in the early stages, the cumulative incidence of cardiovascular events over 3, 6, and 9 years was 13.1%, 15.2%, and 18.5%, respectively, and for those in the advanced stages, it was 22.2%, 31.4%, and 36.5%, respectively.

### Long-term cardiovascular complications

The importance of proactive management measures is highlighted by the wide range of cardiovascular risk factors and long-term consequences that survivors of childhood cancer encounter.

#### Cardiovascular risk factors

Many mechanisms increase the risk of hypertension among survivors of pediatric cancer. In comparison with their peers, these lifestyle characteristics include poorer dietary habits, elevated BMI, lower levels of physical activity, and greater smoking rates [41]. The hypothalamic–pituitary–adrenal axis, sympathetic nervous system, and immune system can all be impacted by psychosocial factors that may raise the risk of hypertension and other cardiovascular diseases. These factors include stress, anxiety, depression, and post-traumatic stress disorder, as well as elevated feelings of social isolation [42]. In addition, early aging carried on by the epigenetic changes brought on by cancer and its treatments can cause cellular senescence and malfunction, which can affect the shape and function of arteries [7].

#### Mitigating risks

Protection measures during and after therapy are necessary since obesity, diabetes mellitus, and hypertension are significant risk factors for AIC [11, 16, 27]. Given its link to coronary heart disease, myocardial infarction, and stroke, hypertension management becomes essential in avoiding early impairment and institutionalization [51]. The prevention and management of cardiovascular diseases heavily depends on medication adherence, blood pressure control, and regular exercise [51].

#### Long-term complications

Survivors face serious cardiovascular risks as a result of cancer treatments, particularly anthracycline chemotherapy and chest-directed radiation therapy [52, 53]. These side effects, which are frequently subtle and silent, can appear years after therapy and put patients at risk for early death or disability [52, 54].

The rapid development of left ventricular (LV) systolic dysfunction and dilated cardiomyopathy (DCM), which can develop silently and suddenly become life-threatening and frequently require urgent medical intervention, is one of the most serious complications [17, 55, 56]. According to reports, the incidence of DCM in children is 0.57 cases per 100,000 per year, with the main cause being anthracycline-induced cardiotoxicity [55]. The permanent death of cardiomyocytes is thought to be the cause of this cardiotoxicity, which results in persistent, progressive heart failure [57, 58]. DCM and LV systolic failure are recognized aftereffects of anthracycline-induced cardiotoxicity that can directly result in hypotension or low blood pressure [17]. Hypotension results from the weakening heart muscle's inability to sustain a healthy blood pressure level. Because it can quickly advance to severe states like cardiogenic shock, when the heart is unable to pump enough blood to the body, potentially resulting in multiple organ failure, this syndrome is especially perilous for young cancer survivors [59]. Hypotension in cancer survivors is not only dangerous at the moment, but it also indicates more serious health issues. It may indicate autonomic dysfunction, which is a prevalent problem in patients with advanced cancer. This can exacerbate symptoms such as tiredness, nausea, and postural hypotension, and may have an adverse effect on survival chances [60]. Hypotension can also be a sign of underlying cardiovascular problems, which increases the risk of heart attacks and strokes—risks that are already higher in cancer survivors [61, 62]. Moreover, the existence of hypotension can result in uncomfortable symptoms including tightness in the chest, breathlessness, and chest pain, which calls for cautious observation and treatment [63]. In conclusion, individuals with cancer, who frequently have advanced age and comparable cardiovascular risk factors, are more vulnerable to orthostatic hypotension. This vulnerability is exacerbated by age-related illnesses and certain drugs, making the management of their health even more challenging [64].

In contrast, the risk of coronary artery disease, heart failure, valve disease, and arrhythmia is greatly increased by hypertension in combination with other cardiovascular risk factors, such as diabetes mellitus, dyslipidemia, and obesity [65].

When contrasting the two conditions, it is important to understand that although both can have major long-term health effects, acute hypotension management is frequently more important because of its potential to cause shock and rapid deterioration, especially in the context of anthracycline-induced cardiotoxicity. However, in order to stop more cardiovascular problems, hypertension needs to be managed over the long term [7, 59, 66].

Less research has been done on the prevalence of hypotension in young cancer survivors than on hypertension. A comprehensive study indicated that when these values were compared to current criteria of systolic hypotension, the clinical cutoffs varied in a way that was consistent with the lower centiles of the population [59]. This data suggests that although hypotension is acknowledged as a clinical problem, its prevalence is not as well defined as that of hypertension, which is 2.6 times more common in pediatric cancer survivors than in the general population [67].

#### Individualized risk assessment

It is critical to optimize modifiable risk factors to reduce the risk of cardiovascular complications, including blood pressure regulation, blood glucose and cholesterol management, weight management, quitting smoking, and medication adherence [10, 68]. More stringent monitoring and preventative interventions, such as the prophylactic use of cardio-protective medicines and routine examinations of cardiac function and structure, may be beneficial for individuals at greater risk [68, 69].

### Screening and monitoring for AIH

The Children's Oncology Group Long-Term Follow-Up Guidelines for Survivors of Childhood, Adolescent, and Young Adult Cancers [5] recommend survivors of childhood cancer to have their blood pressure checked annually by medical specialists. More regular blood pressure checks are recommended for patients who have undergone treatments linked to hypertension, such as corticosteroids, alkylating medications, anthracyclines, or radiation therapy to the chest, abdomen, or kidneys [7].

Blood Pressure Holter Monitoring, also known as ambulatory blood pressure monitoring (ABPM), is a technique for measuring and recording blood pressure that is often used every 15 to 30 min for at least 24 h [70]. When it comes to juvenile cancer survivors who can get late-onset hypertension after chemotherapy, this ongoing observation is really helpful.

One benefit of Blood Pressure Holter Monitoring is that it may provide continuous data, which can be more valuable than single readings obtained at a clinic. This continuous data offers a thorough profile of blood pressure variations during the day and night [71]. It also helps identify hidden hypertension, a condition in which a patient's blood pressure seems normal in a clinical environment but rises during everyday activity [71]. Additionally, it aids in the evaluation of blood pressure circadian rhythms, which is crucial for identifying disorders such nocturnal hypertension, which is linked to an increased risk of cardiovascular disease [71].

Research has indicated that Blood Pressure Holter Monitoring is more effective than clinic measures in predicting blood pressure-related target organ damage, which highlights its usefulness in tracking young cancer survivors [71].

Nevertheless, there are challenges and restrictions, such as the possibility of some kids finding the device uncomfortable, which might affect compliance and data quality. Certain pediatric reference values, which might not be as well established as those for adults, are necessary for data interpretation. The American Heart Association [71] suggests using Blood Pressure Holter Monitoring to confirm hypertension diagnosis in children suspected based on clinic measures. This is one of the recommendations for integrating Holter Monitoring into routine follow-up care.

One important risk factor for AIC is the total amount of anthracycline taken [18]. Consequently, baseline cardiac examinations prior to the first anthracycline dose and continuing assessments throughout therapy, customized to the patient's risk profile and anthracycline type, are advised by the American Society of Clinical Oncology [19]. It is frequently recommended to have echocardiography every 3 months while receiving active treatment, with 1 to 5 years of post-treatment follow-up [72].

First of all, it makes it possible to identify subclinical alterations in heart function before symptoms appear, which allows for prompt intervention [17, 22]. Frequent echocardiographic assessments are crucial for tracking the development of cardiotoxicity over time and determining how well heart failure treatments work [17]. Notably, because echocardiography does not utilize ionizing radiation, it is safe to use repeatedly [73]. Comprehensive echocardiographic results offer important information on cardiac dimensions, such left ventricular dilation, which suggests a loss of cardiomyocytes and eventual heart failure [74]. Right ventricular function can be assessed by echocardiography, which can identify any abnormalities in the ventricle's size, function, or pressure overload brought on by anthracyclines [75]. Furthermore, the assessment of pulmonary artery pressure facilitates the identification of pulmonary hypertension, regardless of its cause—left-sided heart failure or direct cardiotoxicity—thus directing suitable treatment approaches [75]. Additionally, evaluation of left ventricular function aids in identifying impaired cardiac function by taking into account both diastolic and systolic characteristics including flow through the mitral valve and ejection fraction [74]. Systolic dysfunction may be indicated by a decrease in left ventricular ejection fraction (LVEF) below the usual range of 55–70% [76].

The LVEF is still commonly used to measure cardiac function, yet it might not be sensitive enough to identify early cardiac changes brought on by anthracyclines [69]. Greater sensitivity and precision can be obtained by measuring the percentage change in cardiac muscle fiber length during contraction using global longitudinal strain (GLS) [69]. Monitoring for AIC can benefit greatly from GLS since it can identify mild cardiac dysfunction prior to LVEF loss [11]. Furthermore, by demonstrating the degree and stress of myocardial injury and recommending early intervention or therapy modifications, cardiac biomarkers like cardiac troponin and B-type natriuretic peptide can be used in conjunction with echocardiography [11, 77].

Cardio-oncology is a branch of medicine that focuses on identifying, treating, and preventing cardiovascular side effects related to cancer treatment. It includes screening for and monitoring for AIH. The quality of life and long-term outcomes for cancer survivors can be considerably enhanced by identifying those who are at risk, doing routine cardiac evaluations, and putting the right therapies in place.

### Management and intervention strategies

#### Pharmaceutical interventions

Pharmacological treatment seeks to prevent or lessen the cardiotoxic effects of anthracyclines by lowering blood pressure and enhancing heart function. Medication used frequently consists of angiotensin-converting enzyme inhibitors (ACEIs), angiotensin receptor blockers (ARBs), β-blockers, and aldosterone antagonists. Although there is disagreement over the usefulness of these drugs in preventing cardiotoxicity, they have been demonstrated to lower mortality or reverse LV dysfunction in heart failure brought on by chemotherapy [11, 22]. However, since some anti-hypertensive medications may have negative side effects or interact with other medications, it is crucial to monitor the response and tolerance of these medications in childhood cancer survivors and change the dosage or regimen as needed [78].

#### Lifestyle interventions

Lifestyle modifications are essential for lowering blood pressure and enhancing cardiovascular health in addition to medication. Following a balanced diet, exercising frequently, consuming alcohol in moderation, quitting smoking, and maintaining a healthy weight are among the suggestions [29]. With implementing these lifestyle changes, one might lessen the likelihood of AIC while simultaneously reducing the risk of other cardiovascular risk factors [11]. These all-encompassing changes to lifestyle and health management may lower blood pressure by 5 to 10 mmHg and lower the chance of getting new cardiovascular diseases [79].

#### Considerations for survivors with additional cardiovascular risk factors

Before and after cancer treatment, those with a history of heart disease could require more regular cardiac imaging tests, including cardiac magnetic resonance imaging or echocardiography, in order to assess LV function and look for indications of cardiotoxicity [22]. Furthermore, to control blood pressure and other risk factors, they might need more extensive lifestyle and medication interventions [22, 29].

#### Balancing potential adverse effects with effective intervention

Although lifestyle and pharmaceutical interventions have advantages, they are not without restrictions and disadvantages [22, 80]. Certain drugs may have unfavorable interactions with anthracyclines or other cancer treatments, and some lifestyle changes may be difficult to adopt or maintain, particularly for patients receiving intensive cancer treatment or those with low financial means [22, 29, 80]. The benefits and drawbacks of each intervention must thus be carefully weighed, and the treatment strategy must be customized to each patient's unique requirements, preferences, and objectives [22, 29, 80].

### Survivor-centered care and future directions

Employing a survivor-centered approach is essential to provide the best care and support while attending to each survivor's unique needs and preferences.

SCPs are essential documents that detail a survivor's diagnosis, course of therapy, and post-treatment care requirements. Resources for preventative actions, psychological assistance, and details on possible late effects are also included in SCPs [20, 21]. Improved coordination and communication between survivors, oncologists, and primary care physicians is made possible by SCPs, who support survivors in preserving their health and well-being after treatment [20, 21]. To further understand the molecular mechanisms, biomarkers, and genetic variables impacting AIH susceptibility and response, more research is required [81, 82]. We can improve the long-term health outcomes and quality of life for cancer survivors who experience AIH by expanding our knowledge and improving our approaches.

## Conclusions

In conclusion, the remarkable improvements in cancer treatment outcomes may be compromised by AIH, which presents a unique and serious risk to pediatric cancer survivors. A significant number of children, who received anthracycline for cancer treatment, experience long-term cardiovascular side effects, with hypertension being one of the main issues.

Acute, subacute, and chronic cardiotoxicity are the main risks linked to AIH, where hypertension is a side effect of treatment and a trigger for worsening cardiovascular health. The quality of life of survivors is significantly impacted by hypertension, which also increases the risk of heart failure, atherosclerotic cardiovascular disease, ischemic heart disease, and other rhythm abnormalities. Furthermore, cardiomyopathy brought on by anthracyclines is made worse by hypertension.

In order to reduce these risks, it is imperative that hypertension be detected early and that cardiovascular screening be conducted thoroughly. The efficacy of proactive treatment techniques, such as medication and lifestyle adjustments, depends on strong supporting data. Thus, extensive research endeavors are essential to improve prophylactic measures, customize treatments, and expand our comprehension of AIC.

In response, the medical community has to acknowledge and give priority to the distinct cardiovascular dangers that anthracyclines present to children who have survived cancer, while also carrying out more study to clarify the underlying processes. Clinical professionals are unable to ensure that treatment side effects would not shorten the life expectancy provided by cancer survival in the absence of a consistent, evidence-based strategy. We may achieve a balance between treatment efficacy and long-term morbidity by adopting a survivor-centered care approach and embracing transparency, which will eventually improve the quality of life for pediatric cancer survivors.

It is critical to establish exact protocols for the screening, monitoring, and treatment of hypertension in this susceptible group. To include these treatments into routine survivor-ship programs, oncologists, cardiologists, primary care providers, and researchers must work together. This work requires both technical know-how and compassion for the experience of the survivor, so that we are fighting for life but also preserving its quality.

It is time to take action against the growing number of children and young adults who are cancer survivors by stepping up monitoring, improving care, and carrying out thorough research. Together, these actions will protect the long-term health of our youngest cancer survivors, freeing them from the burden of AIH and allowing them to properly celebrate their victories over the disease.

## Data Availability

The data supporting the results presented in this research is accessible through the following electronic databases: PubMed, Google Scholar, and Elsevier. Hyperlinks and persistent identifiers, such as digital object identifiers (DOIs), have been provided. Please refer to the provided references for further details.

## Abbreviations

AIH: Anthracycline-induced hypertension
ECG: Electrocardiogram
DNR: Daunorubicin
AIC: Anthracycline-induced cardiotoxicity
SCPs: Survivor-ship care plan
LV: Left ventricular
DCM: Dilated cardiomyopathy
NO: Nitric oxide
RAAS: Renin–angiotensin–aldosterone system
BMI: Body mass index
VEGF: Vascular endothelial growth factor
LVEF: Left ventricular ejection fraction
GLS: Global longitudinal strain
